# Intra-Uterine Amputations

**Published:** 1903-08-29

**Authors:** 


### INTRA-UTERINE AMPUTATIONS.

In spite of the attention which has been given of recent years to the diseases and deformities of the fœtus, it is somewhat curious that so much uncertainty exists regarding the circumstances which every now and then cause a child to be born minus one or more digits, or the distal part of a limb. It is generally allowed that something of the nature of an amputation has taken place, and indeed the appearance of the stump usually suggests this, but the mechanism by which the amputation is effected is largely a matter of conjecture. Allied to the cases in which there is an actual loss of a member are others in which a groove or constriction is cut more or less deeply into the soft tissues of a limb. It is reasonable to suppose that these are instances of incomplete amputation, and this suggestion is supported by the fact that occasionally, with loss of one member, such a constriction is found on one or other of the remaining limbs. Hence it is in the highest degree probable that both amputations and constrictions result from the action of corresponding agents. The theory that these agents are extra-fœtal bands, arising perhaps from inflammatory adhesions between the cutis and the amnion in an early stage of development, is sometimes opposed on the ground that such amputations occur in cases where no evidence of the existence of these adhesions can be detected. But it must be remembered that these formations, if they occur, are produced at a very early stage, and that there is therefore abundant opportunity for their removal by absorption. The same consideration helps to explain a failure to find the separated digit or limb. Further, there is positive evidence in the occasional discovery of partially separated digits actually surrounded or grasped by bands of lowly organised tissue. A further question which arises for discussion is whether the umbilical cord can possibly act as a constricting or amputating agent. It is certain that the cord is sometimes found in constrictions round the limbs. This is so far *primâ facie* evidence. But it is objected that the cord is too soft a structure to cut through the tissues, and that it may readily slip into grooves formed by some other agency. It must, however, be remembered that in early development the limbs cannot offer anything like firm resistance, and that a continuous strangulation even by a soft ligature may be sufficient to compress the blood-vessels and even to divide the tissues. The balance of evidence seems decidedly to support the view that extra-fœtal bands, including the umbilical cord, are responsible for the occurrence of intra-uterine amputations and constrictions.

